# A Magnesium Based Phosphate Binder Reduces Vascular Calcification without Affecting Bone in Chronic Renal Failure Rats

**DOI:** 10.1371/journal.pone.0107067

**Published:** 2014-09-17

**Authors:** Ellen Neven, Tineke M. De Schutter, Geert Dams, Kristina Gundlach, Sonja Steppan, Janine Büchel, Jutta Passlick-Deetjen, Patrick C. D'Haese, Geert J. Behets

**Affiliations:** 1 Laboratory of Pathophysiology, Department of Biomedical Sciences, University of Antwerp, Antwerp, Belgium; 2 Fresenius Medical Care Deutschland GmbH, Bad Homburg, Germany; 3 Department of Nephrology, University of Düsseldorf, Düsseldorf, Germany; University of Milan, Italy

## Abstract

The alternative phosphate binder calcium acetate/magnesium carbonate (CaMg) effectively reduces hyperphosphatemia, the most important inducer of vascular calcification, in chronic renal failure (CRF). In this study, the effect of low dose CaMg on vascular calcification and possible effects of CaMg on bone turnover, a persistent clinical controversy, were evaluated in chronic renal failure rats. Adenine-induced CRF rats were treated daily with 185 mg/kg CaMg or vehicle for 5 weeks. The aortic calcium content and area% calcification were measured to evaluate the effect of CaMg. To study the effect of CaMg on bone remodeling, rats underwent 5/6th nephrectomy combined with either a normal phosphorus diet or a high phosphorus diet to differentiate between possible bone effects resulting from either CaMg-induced phosphate deficiency or a direct effect of Mg. Vehicle or CaMg was administered at doses of 185 and 375 mg/kg/day for 8 weeks. Bone histomorphometry was performed. Aortic calcium content was significantly reduced by 185 mg/kg/day CaMg. CaMg ameliorated features of hyperparathyroid bone disease. In CRF rats on a normal phosphorus diet, the highest CaMg dose caused an increase in osteoid area due to phosphate depletion. The high phosphorus diet combined with the highest CaMg dose prevented the phosphate depletion and thus the rise in osteoid area. CaMg had no effect on osteoblast/osteoclast or dynamic bone parameters, and did not alter bone Mg levels. CaMg at doses that reduce vascular calcification did not show any harmful effect on bone turnover.

## Introduction

Phosphate binders are routinely used in patients with chronic kidney disease in order to reduce hyperphosphatemia and secondary hyperparathyroidism. As a disturbed phosphate metabolism and abnormal parathyroid hormone (PTH) levels, either low or high, contribute to vascular calcification and renal osteodystrophy, the effect of different phosphate binding agents need to be evaluated on both pathologies. Moreover, increasing evidence for the association between vascular calcification and bone status [1], [2] makes it necessary to investigate changes in the vasculature as well as in bone.

An advanced alternative phosphate binder to pure calcium acetate and pure calcium carbonate is the combined preparation calcium acetate/magnesium carbonate (CaMg, Osvaren) which has the advantage of lowering calcium intake, thereby reducing the risk of soft tissue calcification, while increasing the phosphate binding capacity. In addition, CaMg entails lower medication costs and improved tolerability as compared with calcium-free phosphate binders such as sevelamer hydrochloride (Renagel) and lanthanum carbonate (Fosrenol). The CALMAG study has shown that CaMg is a safe and effective treatment for the regulation of serum phosphorus levels with a good tolerability profile [3]. To study whether this efficient phosphate control leads to beneficial effects in the vasculature, our group investigated the effect of this alternative phosphate binder on vascular calcification in uremic rats. Daily treatment with 750 and 375 mg/kg/day CaMg in adenine-induced uremic rats effectively regulated serum phosphorus and PTH concentrations without inducing any adverse effects on serum calcium levels and significantly reduced the development of aortic calcification [4]. As was the case in the past with aluminum [5], [6] and lanthanum carbonate [7], [8], some concern exists about the potential direct effect of magnesium carbonate on bone remodeling as this alkaline earth metal is absorbed and could possibly interfere with bone mineralization/formation. Although serum magnesium levels were only slightly increased in hemodialysis patients after being treated with CaMg for 24 weeks [3], evaluation of the effect of CaMg therapy on bone is needed.

Following our previous studies with higher doses of CaMg (i.e. 750 and 375 mg/kg/day) [4], in the current study, we investigated whether a 185 mg/kg/day dose of this phosphate binder still inhibits the development of vascular calcification in the rat model with adenine-induced chronic renal failure (CRF). To properly investigate the effects of CaMg on bone parameters, the adenine-induced rat model for CRF, although being highly relevant to study mechanisms and preventive measures of vascular calcification, is less suited to study potential effects on bone. This animal model is characterized by distinctly increased serum phosphorus levels and subsequent severe secondary hyperparathyroidism. Although bone histology indicates a high bone turnover, due to the high amount of woven bone, tetracycline labels are incorporated chaotically and are consequently not measurable which makes accurate analysis of dynamic bone parameters and correct interpretation impossible. For this purpose other models, such as the remnant kidney rat model, are more appropriate. In this model, animals develop a less severe renal failure as compared to animals with adenine-induced uremia resulting in a less severe hyperparathyroid bone allowing measurement of both static and dynamic bone parameters. Although the remnant kidney model does not show vascular calcification without additional interventions (such as high phosphate diet combined with high dose vitamin D), it does allow us to investigate whether CaMg administration at doses sufficient to reduce vascular calcification in adenine-induced CRF rats goes along with effects on bone remodeling. In order to allow us to differentiate between possible phosphate depletion due to the potent pharmacological action of CaMg or a possible direct effect of Mg on bone, rats received either a normal or a high phosphorus diet.

## Material and Methods

### Ethics Statement

Experimental procedures were conducted according to the national Institutes of Health Guide for the Care and Use of Laboratory Animals 85–23 (1996) and approved by the University of Antwerp Ethical Committee (Permit number: 2009–09). Surgery was performed under sodium pentobarbital anesthesia, and all efforts were made to minimize suffering. Animals were monitored daily in order to check health state.

### Effect of a low dose CaMg on vascular calcification - study design

To evaluate whether a relatively low CaMg dose (185 mg/kg/day) is still able to retard the development of aortic calcification, a group of 12 male Wistar rats (250 g, Charles River, Lille, France) was chosen, in which CRF and vascular calcification was induced by feeding a 0.75% adenine/2.5% protein diet (SSNIFF Spezialdiäten, Soest, Germany) for 4 weeks. Prior to CRF induction and after adenine withdrawal, rats were fed a 1.03% phosphorus diet (SSNIFF Spezialdiäten) for 2 weeks. One week after CRF induction, treatment with either vehicle (n = 14) or 185 mg/kg/day CaMg (n = 12) was started by oral gavage in a constant dose volume of 10 ml/kg. After 6 weeks of renal failure animals were sacrificed. Blood and urine samples were taken before CRF induction (week 0), before start of treatment (week 1), after adenine withdrawal (week 4) and at sacrifice (week 6). Rats were sacrificed by exsanguination through the retro-orbital plexus after anaesthesia with intraperitoneal injection of 60 mg/kg pentobarbital.

### Effect of CaMg on bone remodeling - study design

**Figure 1:**
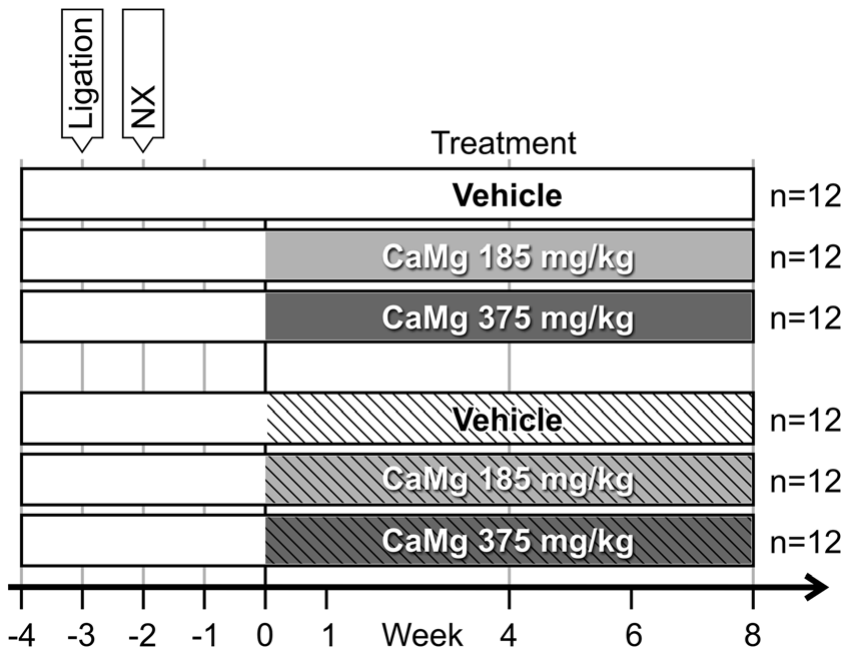
Setup of the study addressing the effect of calcium acetate/magnesium carbonate (CaMg) on bone. The three groups presented in the clear bars received a normal phosphorus diet; those presented in the hatched bars received a high phoshorus diet. Nx: 5/6^th^ nephrectomy.

To evaluate the effect of CaMg on bone in CRF rats, 6 study groups were included, each consisting of 12 male Wistar rats (250 g, Charles River, Lille, France; Figure 1). Hereto, CRF was induced by 5/6^th^ nephrectomy (‘remnant kidney’) which included two steps. First, 2 of the 3 branches of the left renal artery were ligated. One week later, the right kidney was removed. Before surgery, rats were anesthetized by intraperitoneal injection of 60 mg/kg Nembutal (Ceva Santé Animale, Libourne, France). A stabilization period of 2 weeks after nephrectomy was respected before the start of treatment.

The first three CRF groups were maintained on a regular rodent diet with normal phosphorus content (0.8% P), the next three groups were fed a high phosphorus diet (1.03% P) (SSNIFF Spezialdiäten). Thus, the expectation was to distinguish indirect effects of CaMg treatment (i.e. phosphate depletion due to the high phosphate binding capacity of the compound) from direct effects of magnesium on bone. CRF animals were assigned to different treatment groups: (1) Vehicle, (2) 185 mg/kg CaMg or (3) 375 mg/kg CaMg. CaMg was dissolved in 1% carboxymethylcellulose and administered daily by oral gavage in a constant dose volume of 10 ml/kg for 8 weeks.

Blood and urine samples were taken every 4 weeks throughout the study period, i.e. at baseline (week −4, before surgery), at week 0 (before treatment), at week 4 and 8 (sacrifice). Animals were maintained for 24 h in metabolic cages to collect urine and subsequently, blood was drawn by puncture of the tail vein.

Animals were given 30 mg/kg doses of tetracycline and demeclocycline at 7 and 3 days before sacrifice to allow measurement of dynamic histomorphometric bone parameters. After 8 weeks of CRF, animals were sacrificed by exsanguination through the retro-orbital plexus after anaesthesia with intraperitoneal injection of 60 mg/kg pentobarbital.

### Biochemical analyses

Serum creatinine was determined according to the Jaffé method. Serum ionized calcium was measured with iSTAT (Abott Diagnostics, Belgium). Magnesium and total calcium in serum and urine were measured with flame atomic absorption spectrometry (Perkin-Elmer, Wellesley, MA, USA) after appropriate dilution in 0.1% La(NO_3_)_3_. Serum PTH was determined with the rat intact PTH ELISA kit (Immutopics Inc, San Clemente, CA, USA). Serum and urinary phosphorus were measured using the EcolineS Phosphate kit (DiaSys, Germany).

### Evaluation of vascular calcification

After isolation of the thoracic aorta of the adenine-induced CRF rats, tissue was fixed in neutral buffered formalin and cut into sections of 2–3 mm. These sections were embedded upright in a paraffin block and a 4 µm section was stained for vascular calcification with Von Kossa's method and counterstained with haematoxylin and eosin. The% of calcified area was calculated using Axiovision image analysis software (Release 4.5, Carl Zeiss, Oberkochen, Germany) in which two color separation thresholds measure the total tissue area and the Von Kossa positive area. After summing both absolute areas, the% of calcified area was calculated as the ratio of the Von Kossa positive area versus the total tissue area.

The proximal part of the abdominal aorta was isolated and weighed on a precision scale. Subsequently, samples were digested in 65% HNO_3_ at 60°C overnight. The calcium and magnesium content of the tissue was measured with flame atomic absorption spectrometry and expressed as mg calcium or magnesium/g wet tissue.

### Bone analyses

Both tibia of the 5/6^th^ nephrectomized rats were isolated and cleared from surrounding tissue. After fixation in 70% ethanol overnight, the left tibia was further processed for bone histomophometric analysis: tibias were dehydrated, and embedded in 100% methylmetacrylate (Merck, Hohenbrunn, Germany). Five µm thick sections were Goldner stained for visualization and measurement of mineralized bone, osteoid and eroded perimeter which were quantified with Axiovision image analysis software (Release 4.5, Carl Zeiss, Oberkochen, Germany). Ten µm thick unstained sections of the tibia were used to measure tetracycline labels by fluorescence microscopy. Out of these primary data, additional histological bone parameters and dynamic bone parameters are calculated according to Parfitt et al. [9].

The right tibia was isolated and its wet weight was recorded. Tibia samples were digested in 65% HNO_3_ at 60°C overnight. Flame atomic absorption spectrometry was used to determine the calcium and magnesium content in bone samples.

### Statistics

Results are expressed as mean ± standard deviation unless otherwise indicated. Non-parametric statistical analyses were performed with SPSS 20.0 software. Statistical differences between groups were investigated with Kruskall-Wallis test followed by Mann Whitney-U test. Comparison of time-points was performed with a Friedman related samples test, followed by a Wilcoxon signed ranks test. Bonferroni correction was applied when appropriate and p<0.05 was considered significant.

## Results

### Effect of a low dose CaMg on vascular calcification

#### Biochemical results

The effect of a low CaMg dose (185 mg/kg/day) on vascular calcification was investigated in adenine-induced CRF animals. Renal function decreased progressively after start of adenine feeding as indicated by a significant 5 to 10-fold increase in serum creatinine levels in both vehicle and CaMg treated CRF groups (data not shown). Induction of severe CRF by adenine administration decreased ionized serum calcium levels in both groups (Table 1). Daily gavage with 185 mg/kg CaMg did not increase ionized calcium in the serum. Due to the renal impairment, manifest hyperphosphatemia was seen from week 4 onwards, which was then prevented by treatment with 185 mg/kg/day CaMg. Magnesium levels were slightly increased after 4 weeks of CRF in CaMg animals; they remained modestly elevated versus vehicle treated controls until sacrifice.

**Table 1:** Serum biochemistry of vehicle versus 185 mg/kg CaMg treated adenine-induced CRF rats.

Group	Week 0	Week 1	Week 4	Week 6
**Serum Ionized Calcium (mg/dl)**				
Vehicle	5.20±0.20	4.94±0.08°	4.39±0.34°	4.23±0.59°
185 CaMg	5.11±0.09	4.79±0.18°*	4.42±0.18°	4.48±0.42°
**Serum Phosphorus (mg/dl)**				
Vehicle	6.21±0.56	4.82±0.99°	20.34±12.01°	8.91±2.19°
185 CaMg	6.54±0.94	6.15±1.91	8.95±1.53°*	7.15±1.23*
**Serum Magnesium (mg/dl)**				
Vehicle	1.74±0.05	1.70±0.09	2.80±0.28°	2.42±0.25°
185 CaMg	2.02±0.11*	2.20±0.14°*	4.21±0.28°*	3.21±0.27°*

*: p<0.05 vs. vehicle treated CRF rats, same time point.

°: p<0.05 vs. week 0, same treatment group.

#### Vascular calcification

**Figure 2:**
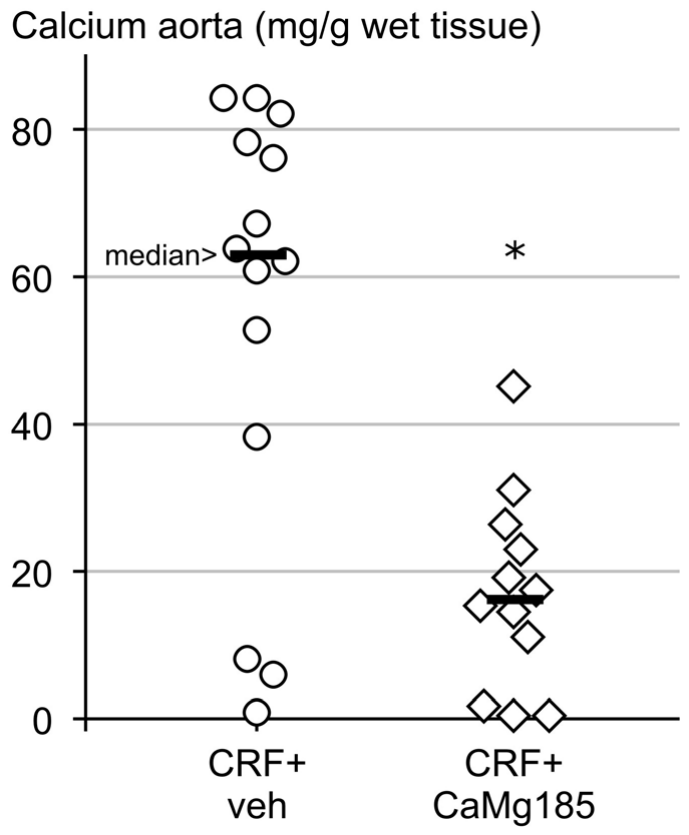
Aortic calcification in vehicle versus 185 mg/kg CaMg treated chronic renal failure (CRF) rats. *: p<0.05 vs. CRF + vehicle.

Adenine-induced CRF caused severe calcification in the aorta as shown by the excessive calcium concentration in the abdominal aorta (Figure 2). Daily treatment with 185 mg/kg CaMg significantly reduced the aortic calcium content of CRF rats by approximately 75%. However, the dose of 185 mg/kg CaMg did not significantly decrease the area% calcification in the thoracic aorta as compared with vehicle treated CRF animals (27±10% in the vehicle group versus 23±14% in the CaMg group).

### Effect of CaMg on bone remodeling

#### Biochemical results

The effect of CaMg on bone turnover was evaluated in 5/6^th^ nephrectomized animals receiving normal or high phosphorus diet. Renal impairment was present in all 5/6^th^ nephrectomized animals at week 0 (start of CaMg treatment i.e. 2 weeks after final surgery) as indicated by a doubling of serum creatinine levels and remained constant throughout the treatment period (data not shown). Neither the diet nor CaMg treatment had an effect on renal function of CRF rats.

During the study period, total serum calcium levels tended to be higher towards the end of the study, which was statistically significant after 8 weeks in most groups, but no significant differences between treatment groups were found at the same time point (data not shown). Daily treatment with both CaMg doses did not cause major differences in serum ionized calcium levels (Table 2). Urinary calcium excretion increased in all groups as a result of renal failure (Table 3). This effect was stronger in vehicle treated animals receiving normal phosphorus diet than in vehicle animals receiving the high phosphorus diet, resulting in significantly lower calcium excretion in 24 h in the latter group. No significant differences were found between CaMg and vehicle treated rats.

**Table 2:** Serum biochemistry over time of 5/6^th^ nephrectomized rats receiving normal phosphorus (NP) or high phosphorus (HP) diet, in combination with vehicle (Veh), 185 or 375 mg/kg CaMg.

Group	Week −4	Week 0	Week 4	Week 8
**Serum Ionized Calcium (mg/dl)**				
NP, Veh	5.04±0.20	4.84±0.29	4.93±0.18	4.68±0.50
NP, 185	5.13±0.15	4.80±0.26	4.85±0.27	4.96±0.13
NP, 375	5.15±0.11	4.98±0.18	4.88±0.20	4.97±0.17
HP, Veh	5.14±0.16	4.86±0.29	4.43±0.73	4.97±0.27
HP, 185	5.06±0.15	4.89±0.20	4.55±0.43	4.62±0.48
HP, 375	5.12±0.12	4.78±0.53	4.61±0.43	5.05±0.30
**Serum Phosphorus (mg/dl)**				
NP, Veh	7.74±1.42	8.77±1.74	7.36±1.42	9.22±3.07
NP, 185	7.66±2.11	6.09±0.89	6.17±0.99	8.38±3.73
NP, 375	8.02±0.94	7.16±1.77	6.83±1.12	11.91±4.13°
HP, Veh	7.85±1.59	7.34±2.05	6.18±2.00	7.39±2.39
HP, 185	8.38±1.42	7.27±0.98	6.45±1.72	12.99±9.30
HP, 375	7.81±0.94	6.43±1.05	6.76±1.10	9.77±3.59
**Serum Magnesium (mg/dl)**				
NP, Veh	2.25±0.13	2.54±0.25	2.74±0.32°	3.44±0.72°
NP, 185	2.37±0.12	2.79±0.15	2.74±0.23°	3.40±0.71°
NP, 375	2.22±0.12	2.67±0.33	2.76±0.56°	3.18±0.40°
HP, Veh	2.27±0.18	2.53±0.21	2.37±0.28	2.51±0.22 *
HP, 185	2.16±0.27	2.46±0.24	2.49±0.21	3.45±1.27°
HP, 375	2.22±0.32	2.42±0.12	2.40±0.16	2.86±0.54°
**Serum PTH (pg/ml)**				
NP, Veh	826±1335			2792±1866°
NP, 185	578±314			2140±797°
NP, 375	504±221			2902±2788°
HP, Veh	564±158			5572±6416°
HP, 185	595±580			6869±5384°
HP, 375	668±166			4675±2915°

*: p<0.05 vs NP, same dose.

°: p<0.05 vs week −4, same diet and dose.

**Table 3:** Urine biochemistry over time of 5/6^th^ nephrectomized rats receiving normal phosphorus (NP) or high phosphorus (HP) diet, in combination with vehicle (Veh), 185 or 375 mg/kg CaMg.

Group	Week −4	Week 0	Week 4	Week 8
**Urinary Calcium (mg/24 h)**				
NP, Veh	1.5±1.1	8.1±3.4°	9.9±7.3°	4.9±2.7°
NP, 185	1.7±1.1	10.5±6.1°	10.6±5.8°	5.4±2.8
NP, 375	1.7±1.6	8.2±2.8°	8.9±10.1°	4.8±2.7
HP, Veh	1.4±0.5	5.0±2.7°	4.1±2.0 *°	3.0±1.1
HP, 185	1.5±0.4	6.9±5.9°	5.9±2.5°	3.6±1.8
HP, 375	1.9±0.7	5.0±3.6°	8.5±7.2°	4.3±2.8
**Urinary Magnesium (mg/24 h)**				
NP, Veh	1.9±0.5	7.7±2.1°	8.5±2.7°	7.2±3.0°
NP, 185	2.6±1.0	7.8±1.5°	8.3±1.7°	5.5±1.4°
NP, 375	1.5±0.5	6.4±3.1°	6.2±1.3°	6.4±1.4°
HP, Veh	3.4±0.7 *	6.6±1.3°	5.8±2.7°	5.3±0.5
HP, 185	3.0±0.6	7.8±2.0°	7.3±3.2°	5.3±3.8
HP, 375	3.3±0.9 *	6.4±1.7°	9.1±1.7 *°	8.3±3.5

*: p<0.05 vs NP, same dose.

°: p<0.05 vs week −4, same diet and dose.

**Figure 3:**
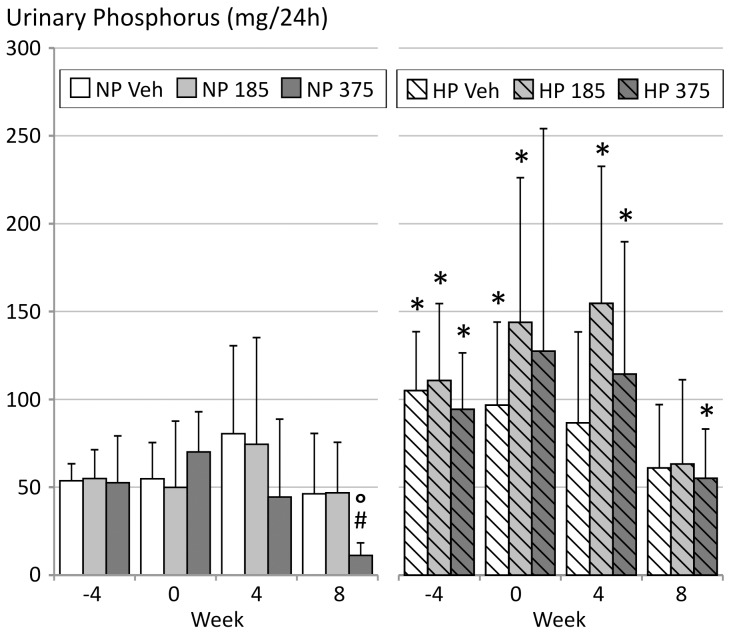
Urinary phosphorus levels in 5/6^th^ nephrectomized rats on a normal (NP) or high phosphorus (HP) diet treated with either vehicle (veh), 185 or 375 mg/kg/day CaMg. *: p<0.05 vs NP, same dose; #: p<0.05 vs vehicle, same diet; °: p<0.05 vs week −4, same diet and dose.

After 8 weeks of renal failure, serum phosphorus levels tended to be higher in all study groups, although a statistical significance was only reached in animals receiving a normal phosphorus diet and 375 mg/kg/day CaMg (Table 2). These animals also showed a significant decrease of urinary phosphorus excretion versus vehicle after 8 weeks of treatment (Figure 3). In the groups receiving a high phosphorus diet, phosphorus excretion was significantly higher than in the animals receiving the normal phosphorus diet, but no significant differences between CaMg doses or vehicle were observed (Figure 3).

Serum magnesium levels slightly increased in all groups during treatment (Table 2). Also urinary magnesium excretion was increased in all groups during treatment, following a similar pattern as urinary calcium excretion. However, no significant differences between CaMg treated groups were found (Table 3).

Due to renal failure, serum PTH values were significantly elevated at the end of the study in all groups, however, no difference between groups was noted (Table 3). Rats on a high phosphorus diet tended to show higher serum PTH concentrations in comparison with those on a normal phosphorus diet.

#### Bone

**Figure 4:**
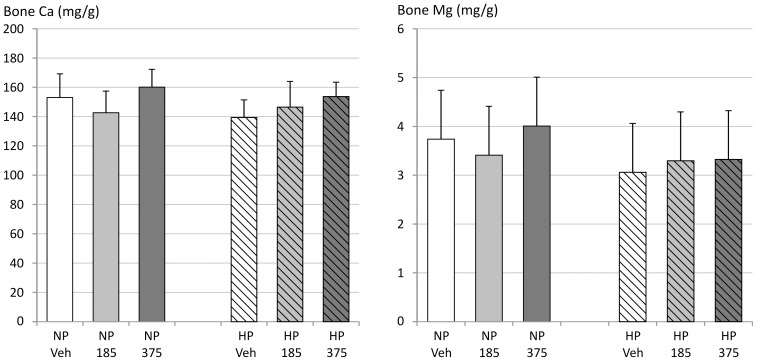
Calcium and magnesium content in the tibia of 5/6^th^ nephrectomized rats on a normal (NP) or high phosphorus (HP) diet treated with either vehicle (veh), 185 or 375 mg/kg/day CaMg.

**Figure 5:**
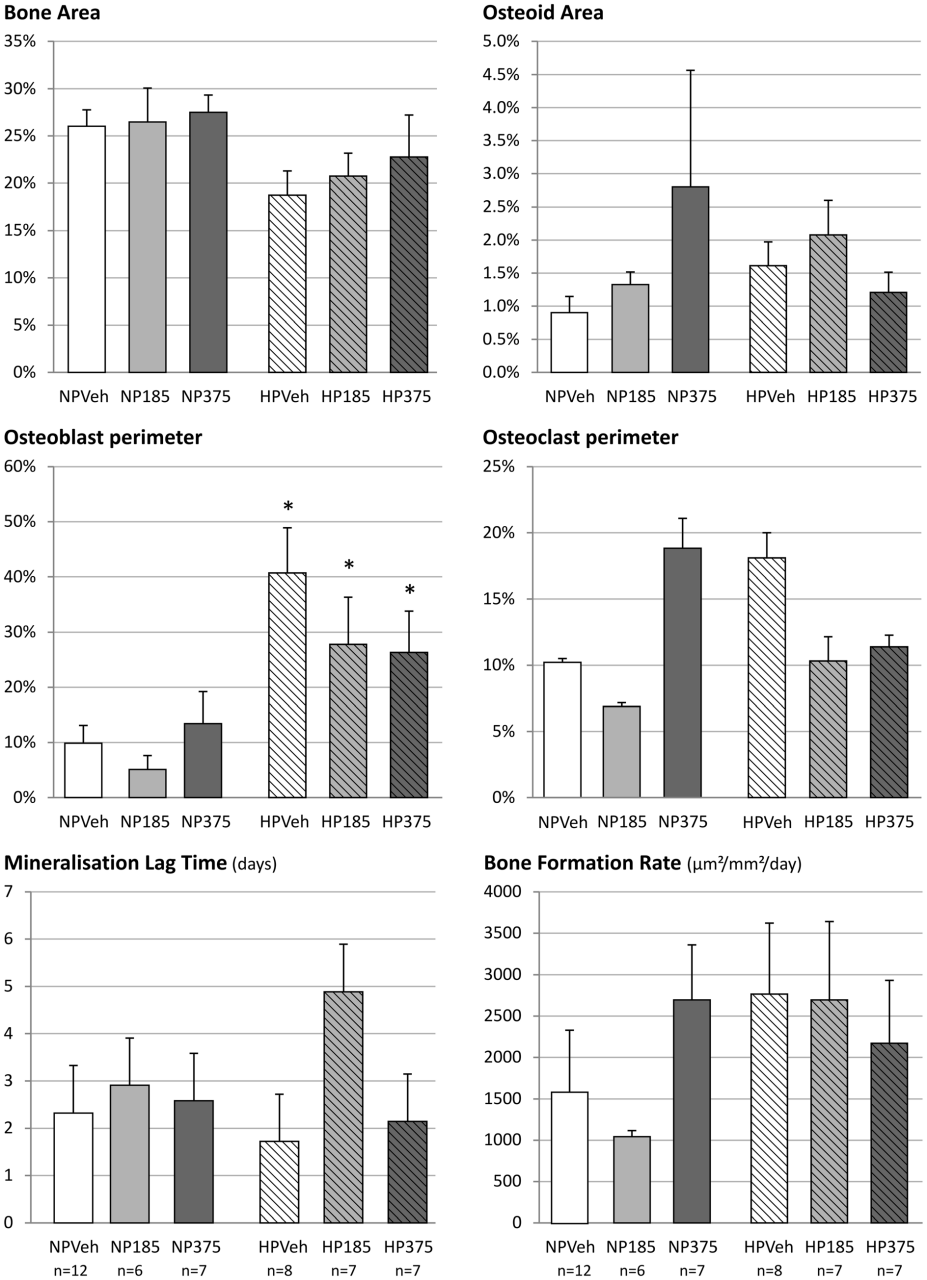
Static and dynamic bone parameters of 5/6^th^ nephrectomized rats on a normal (NP) or high phosphorus (HP) diet treated with either vehicle (veh), 185 or 375 mg/kg/day CaMg. *: p<0.05 vs. NP, same dose.

Calcium as well as magnesium concentrations in bone showed no significant differences between vehicle and groups at CaMg doses able to reduce vascular calcification (Figure 4). The Ca/Mg ratio also did not show any significant differences (results not shown). Bone histomorphometric analysis revealed that bone area was not significantly different between groups (Figure 5). Osteoid area and osteoclast perimeter tended to increase, while osteoblast perimeter was increased significantly in vehicle-treated animals on a high phosphorus diet in comparison with those on a standard phosphorus diet (Figure 5). In the animals receiving the high phosphorus diet treated with CaMg, these changes were less pronounced, especially in the animals receiving the highest dose.

**Figure 6:**
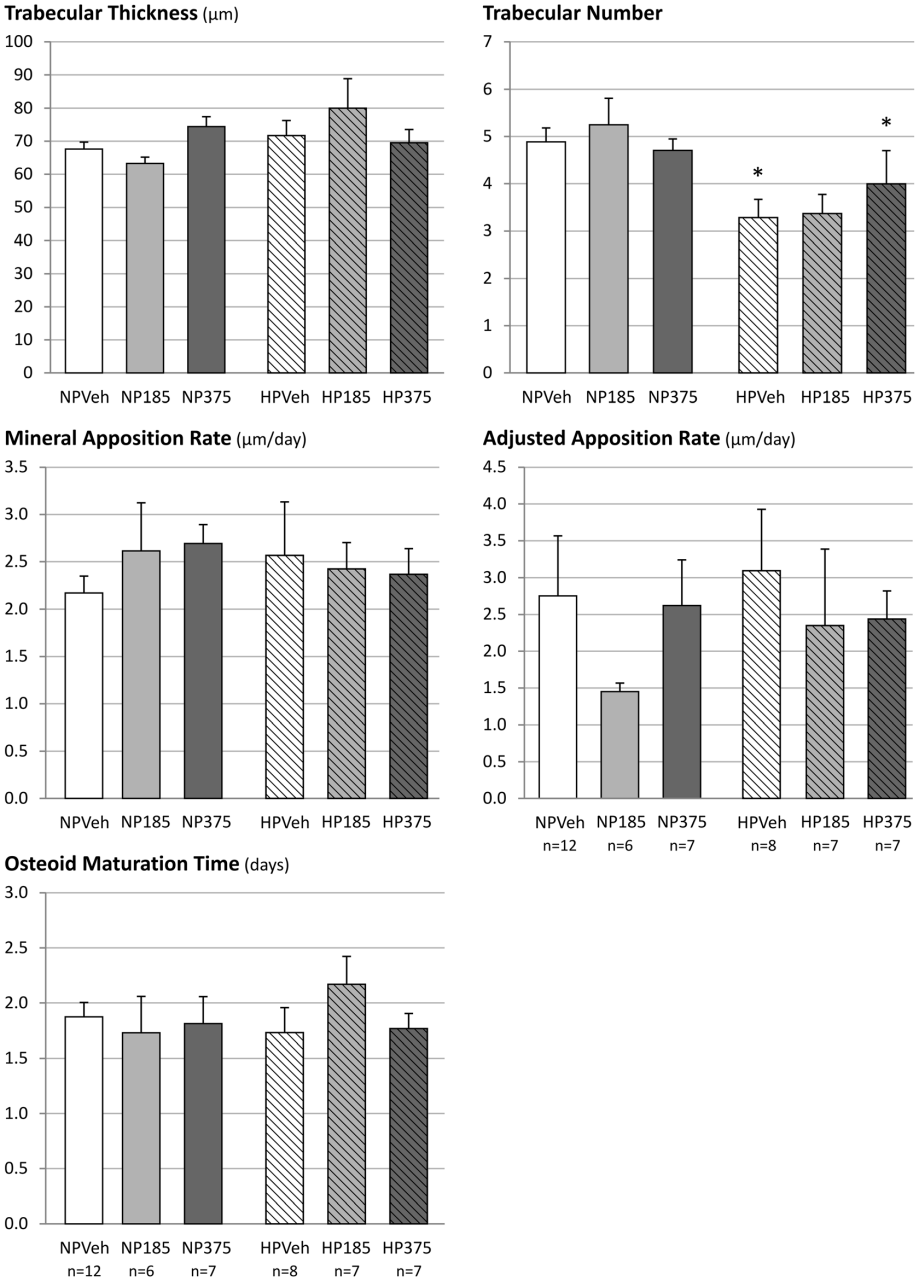
Static and dynamic bone parameters of 5/6^th^ nephrectomized rats on a normal (NP) or high phosphorus (HP) diet treated with either vehicle (veh), 185 or 37 mg/kg/day CaMg. *: p<0.05 vs. NP, same dose.

Osteoid area showed a CaMg dose-dependent increase in animals receiving the normal phosphorus diet, which was accompanied with an increased osteoclast perimeter, although statistical significance was not reached. Trabecular numbers were significantly lower in animals receiving high phosphorus diet treated with either vehicle or CaMg 375 mg as compared to those on a normal phosphorus diet, but no changes were seen in the trabecular thickness (Figure 6). Dynamic bone parameters such as bone formation rate, mineralization lag time, osteoid maturation time, mineral apposition rate and adjusted apposition rate showed no consistent changes with treatment or diet (Figures 5 and 6).

## Discussion

The main concern regarding the new phosphate binder CaMg is a possible harmful effect of magnesium (alkaline earth metal) on bone turnover and mineralization as was the concern with the introduction of lanthanum carbonate, a metal containing phosphate binder. More specifically, the question arose whether treatment with CaMg in chronic kidney disease might cause low turnover bone disease; i.e. osteomalacia or adynamic bone, which in the past has been seen with aluminum [10]–[12]. These effects were due to a direct interference of aluminum with bone mineralization, a direct effect on the osteoblast and the parathyroid glands [13]–[16]. In the present study, bone histomorphometric analyses were performed in CRF animals treated with CaMg to address this question.

We have previously shown that using adenine-induced CRF rats, doses of 750 and 375 mg/kg CaMg were able to effectively control serum phosphorus levels and to significantly inhibit the development of vascular calcifications [4]. That study also revealed that the reduction in aortic calcification with this phosphate binder did not coincide with an altered expression of osteochondrogenic markers in the vessel wall. The same rat model was used in the current study to investigate whether a lower dose of 185 mg/kg CaMg could still inhibit calcification in the vessel wall. Measurement of calcium content in the aorta, which is a very sensitive method to quantify tissue calcification, indeed confirmed that CaMg treatment at this dose significantly reduced aortic calcification in rats with severe CRF. Evaluation of the percentage calcified surface on Von Kossa stained aortic cross-sections revealed no difference between vehicle and 185 mg/kg CaMg treatment in CRF animals. These results indicate that the density of the aortic calcification was distinctly regressed by a daily dose of 185 mg/kg CaMg, which, however, cannot be observed when only the surface of the calcified lesion in the arterial wall, as measured by the area% calcification (Von Kossa positivity), is taken into account. Our previous study revealed that the higher CaMg doses (375 and 750 mg/kg) reduced both the calcium content and the area% calcification in the aorta.

Despite the beneficial effect of relatively low doses of CaMg on vascular calcification, our previous experiment performed in adenine-induced CRF rats treated with 750 and 375 mg/kg CaMg revealed an increased amount of osteoid in the bone. This observation was hypothesized to be due to a phosphate depletion in these animals, which results from the high phosphate binding capacity of CaMg, leading to either an increased efflux of phosphate (and calcium) from the bone and/or a decreased incorporation of both minerals into the bone. However, a direct effect of magnesium on bone mineralization could not be excluded. In order to further investigate this hypothesis, measurements of both static and dynamic bone parameters are required. The adenine-induced renal failure model is less suited for studying bone turnover since it does not allow accurate measurement of bone dynamics after tetracycline labeling. Therefore, in the current study, a milder model of renal failure, i.e. the 5/6^th^ nephrectomy rat model which is a well characterized and valuable animal model for the study of renal bone disease [17]–[19], was used. The animals were treated with 375 or 185 mg/kg/day CaMg at high or normal phosphorus diet. As shown previously, these doses are able to reduce vascular calcification in CRF.

Bone histomorphometric analysis showed a slight increase in osteoid area in CRF rats on a normal phosphorus diet receiving the highest CaMg dose which is one of the histological features of osteomalacia. However, whereas in pure (aluminum-induced) osteomalacia the increased osteoid goes along with a distinctly decreased number of osteoblasts and osteoclasts, a dramatically reduced bone formation rate and mineral apposition rate as well as an increased osteoid maturation time, in the present study all these parameters were not affected, indicating that the increased amount of osteoid seen in this study group must be due to another factor. While the low dose of 185 mg/kg/day did not induce a noticeable decrease in urinary phosphorus excretion, treatment with high dose CaMg induced a marked drop in urinary phosphorus excretion in the group receiving the normal phosphorus diet. This points to a phosphate depletion due to the potent phosphate binding of this compound. This phosphate depletion could in turn disturb mineralization, leading to an increased amount of osteoid, while not directly affecting the number of osteoblasts or osteoclasts. Similar observations were also made in the past with other powerful phosphate binding agents such as lanthanum carbonate and sevelamer [17], [20]. Moreover, in the animals receiving the high phosphorus diet, no effect of CaMg on urinary phosphorus excretion compared to vehicle was seen, which indicates that the higher phosphorus content of the diet prevented phosphate depletion and thus osteoid accumulation. This supports the statement that phosphate depletion is the most likely cause for the increased osteoid area. Moreover, as dynamic bone parameters such as mineralization rate and osteoid maturation time were not altered, a direct interference of magnesium with bone mineralization leading to osteomalacia can be excluded.

Furthermore, the data also provide no evidence that CaMg induces adynamic bone disease, since treatment with this agent did neither affect the number of osteoblasts and osteoclasts nor the bone formation rate, two hallmarks of adynamic bone. In view of this, a post hoc evaluation of the CALMAG study [3] recently showed that CaMg treatment in hemodialysis patients did not influence markers of bone turnover [21].

Lastly, there is no indication of accumulation of magnesium in the animals with the doses sufficient to efficiently reduce vascular calcification as no increased magnesium levels were found in the bone.

The absence of a significant rise in serum and bone magnesium levels at these doses is not surprising in view of the mild CRF and the systemic regulatory mechanisms that exist with regard to the absorption/excretion of the element [22]. Even in the adenine-induced CRF rat representing a model for severe renal impairment, bone magnesium concentrations were not significantly increased in CRF rats treated with a relatively high 750 mg/kg CaMg dose as compared to vehicle treated ones, despite significant elevations in serum magnesium levels up to 7 mg/dl (own unpublished results).

The treatment and effective phosphate binding, however, was proven and had an impact in the high phosphorus diet group: administration of this diet in vehicle treated animals resulted in hyperparathyroid bone disease as indicated by the substantial rise in osteoid area, osteoblast and osteoclast number and bone formation rate as well as a reduction of the bone area and trabecular number, in combination with higher serum PTH levels when compared to vehicle treated animals receiving the normal phosphorus dose. Treatment with CaMg in the high phosphorus fed groups dose-dependently ameliorated these static and dynamic bone parameters due to effective phosphate binding. Taken together the data enlighten the ongoing discussion about possible effects of magnesium on bone health and homeostasis. In the current study, CRF rats were treated with CaMg doses similar to clinical doses in dialysis patients without any negative changes in bone metabolism. The combined preparation calcium acetate/magnesium carbonate is thus a valuable, safe and effective, alternative for calcium containing phosphate binders on the one hand, which should be prescribed with caution in patients with adynamic bone disease for the risk of stimulating vascular calcification, and lanthanum carbonate and sevelamer carbonate on the other hand which are much more expensive in comparison to CaMg.

In conclusion, CaMg treatment at doses that reduce vascular calcification ameliorated high bone turnover parameters in 5/6^th^ nephrectomized rats on a high phosphorus diet and did not cause an accumulation of magnesium in the bone. The increased osteoid area in the 375 mg/kg treated CRF group receiving a normal phosphorus diet is most likely due to the phosphate depletion resulting from the high phosphate binding potential of CaMg, rather than being the consequence of a direct magnesium effect on bone. Thus, CaMg treatment did not adversely affect bone remodeling.
